# PCR colorimetric dot-blot assay and clinical pretest probability for diagnosis of Pulmonary Tuberculosis in Smear-Negative patients

**DOI:** 10.1186/1471-2458-7-356

**Published:** 2007-12-20

**Authors:** Luciene Cardoso Scherer, Rosa Dea Sperhacke, Carla Jarczewski, Patrícia I Cafrune, Simone Minghelli, Marta Osório Ribeiro, Fernanda CQ Mello, Antonio Ruffino-Netto, Maria LR Rossetti, Afrânio L Kritski

**Affiliations:** 1 Programa de pós Graduação em Ciências Biológicas- Bioquímica, Universidade Federal do Rio Grande do Sul-UFRGS, Porto Alegre, RS, Brazil; 2 Centro de Desenvolvimento de Ciência e Tecnologia- CDCT, Fundação Estadual de Produção e Pesquisa em Saúde-FEPPS/RS, Porto Alegre, RS, Brazil; 3 Universidade Luterana do Brasil-ULBRA, Canoas, RS, Brazil; 4 Unidade de Pesquisa em Tuberculose, Hospital Universitário Clementino Fraga Filho, UPT/HUCFF, Universidade Federal do Rio de Janeiro-UFRJ, Rio de Janeiro, RJ, Brazil; 5 Hospital Sanatório Partenon/Secretaria da Saúde do Rio Grande do Sul, Porto Alegre, RS, Brazil; 6 State Reference Laboratory (Laboratório Central do Rio Grande do Sul- Lacen, RS), Fundação Estadual de Produção e Pesquisa em Saúde-FEPPS/RS) Porto Alegre, RS, Brazil; 7 Faculdade de Medicina de Ribeirão Preto, Universidade de São Paulo, SP, Brazil

## Abstract

**Background:**

Smear-negative pulmonary tuberculosis (SNPTB) accounts for 30% of Pulmonary Tuberculosis (PTB) cases reported annually in developing nations. Polymerase chain reaction (PCR) may provide an alternative for the rapid detection of *Mycobacterium tuberculosis *(MTB); however little data are available regarding the clinical utility of PCR in SNPTB, in a setting with a high burden of TB/HIV co-infection.

**Methods:**

To evaluate the performance of the PCR dot-blot in parallel with pretest probability (Clinical Suspicion) in patients suspected of having SNPTB, a prospective study of 213 individuals with clinical and radiological suspicion of SNPTB was carried out from May 2003 to May 2004, in a TB/HIV reference hospital. Respiratory specialists estimated the pretest probability of active disease into high, intermediate, low categories. Expectorated sputum was examined by direct microscopy (Ziehl-Neelsen staining), culture (Lowenstein Jensen) and PCR dot-blot. Gold standard was based on culture positivity combined with the clinical definition of PTB.

**Results:**

In smear-negative and HIV subjects, active PTB was diagnosed in 28.4% (43/151) and 42.2% (19/45), respectively. In the high, intermediate and low pretest probability categories active PTB was diagnosed in 67.4% (31/46), 24% (6/25), 7.5% (6/80), respectively. PCR had sensitivity of 65% (CI 95%: 50%–78%) and specificity of 83% (CI 95%: 75%–89%). There was no difference in the sensitivity of PCR in relation to HIV status. PCR sensitivity and specificity among non-previously TB treated and those treated in the past were, respectively: 69%, 43%, 85% and 80%. The high pretest probability, when used as a diagnostic test, had sensitivity of 72% (CI 95%:57%–84%) and specificity of 86% (CI 95%:78%–92%). Using the PCR dot-blot in parallel with high pretest probability as a diagnostic test, sensitivity, specificity, positive and negative predictive values were: 90%, 71%, 75%, and 88%, respectively. Among non-previously TB treated and HIV subjects, this approach had sensitivity, specificity, positive and negative predictive values of 91%, 79%, 81%, 90%, and 90%, 65%, 72%, 88%, respectively.

**Conclusion:**

PCR dot-blot associated with a high clinical suspicion may provide an important contribution to the diagnosis of SNPTB mainly in patients that have not been previously treated attended at a TB/HIV reference hospital.

## Background

Tuberculosis (TB) is one of the most widespread mortality-causing infectious diseases in humans. Timely detection of the disease allows the institution of an effective and life-saving treatment, thereby reducing transmission to close contacts. Conventional diagnosis of Pulmonary Tuberculosis (PTB) is time-consuming, and the acid fast bacilli (AFB) smear has a low sensitivity (40%–60%) [1]. HIV infection has been associated with an increased incidence of smear negative pulmonary tuberculosis (SNPTB) and a higher mortality rate in TB patients. [2-4]. In Brazil, almost 30% of PTB cases among adults are SNPTB [2-4]. Diagnosis of SNPTB is a difficult task and, in developing countries, the majority of these cases have been treated only on the basis of clinical and chest radiographic findings. Without a standardized clinical work up, the misdiagnosis rates have been estimated to be as high as 35% [5]. Therefore, in settings with a high rate of TB and HIV, the clinical evaluation of new tools for smear negative PTB diagnoses is extremely valuable [1,6]

In industrialized countries, tests for *Mycobacterium tuberculosis *(MTB), using rapid nucleic acid amplification (NAA), have been considered a major breakthrough in the diagnosis of PTB [7]. In developing countries, the *in house *polymerase chain reaction (PCR) for amplification of MTB DNA, using the IS*6110 *insertion element as a target, offers a potentially sensitive, specific and low-cost test that could provide a rapid diagnosis of PTB [8-11].

In these settings, the published evaluations of NAA techniques for smear negative PTB diagnosis have been based mainly on laboratory criteria for diagnosis of disease with or without clinical records used to evaluate discrepant results [11-17].

In the present study, we investigated the performance of a home-made colorimetric PCR (PCR dot-blot) to diagnose TB using expectorated sputum from patients suspected of having SNPTB, in isolation and in parallel with pretest probability (based on Clinical Suspicion) in a hospital setting with a high burden of TB and HIV. The PCR technique performance was compared with conventional routine diagnostic methods for smear negative patients.

## Methods

### Setting and patient selection

Consecutive adults suspected of having SNPTB, referred to the TB and HIV Reference Center, Parthenon Reference Hospital (PRH) in Porto Alegre City, capital of Rio Grande do Sul, State of Brazil, were studied prospectively, from May 2003 to May 2004. SNPTB suspects were referred from community health care units to have their respiratory specimens cultured for mycobacteria, according to Brazilian National Guidelines [18].

Eligible patients were those: (1) who reported more than 3 weeks of cough; (2) who had two consecutive samples of spontaneous sputum that were acid fast bacilli smear-negative. Patients illegible were those receiving anti-TB treatment. Patients with a history of previous TB were not excluded. Patients were excluded from the study if any of the following conditions were met: (1) culture was contaminated; (2) when expectorated sputum was not obtained (3) laboratory or clinical data did not fulfill the SNPTB definition; (4) written informed consent was not obtained from the study participant. All clinical samples were sent to the Laboratory of the State of RS, State Foundation for Research in Health, Porto Alegre/RS/Brazil, (FEEPS/Lacen/RS) for laboratory analysis. This study was approved by the Institutional Review Boards of FEPPS/RS (n. 01/2002).

Suspects of SNPTB, after signing their written informed consent, underwent a validated questionnaire with questions regarding demographic variables and clinical history (e.g.: smoking, alcohol abuse, HIV infection/AIDS)[19]. Chest radiographs and physical examination was performed by a respiratory specialist using a standardized form. Respiratory specialists were blinded for the results of cultures and PCR dot-blot, and laboratory technicians were blinded for the chest radiographs results and clinical predictors. HIV testing by ELISA was performed, using Western blot as a confirmatory test.

### Estimate of pretest probability

To estimate pretest probability (clinical suspicion), all eligible individuals were classified into three relative risk categories during the first appointment: low (≤ 25%); intermediate (26%–75%); and high (>75%) pretest probabilities of active PTB. Classifications were made by four respiratory specialists (2 with 10 years of experience and 2 with 20 years of experience in diagnosing TB). This was an estimate of disease probability based on clinical history, physical examination, other laboratory data available besides microbiological tests, and chest radiographs evaluation performed using a validated form [19].

### Radiographic analysis

Chest radiographs were classified as typical, compatible, atypical and normal. Typical were those considered as having any parenchymal infiltrate or cavity localized in the upper zone (defined as the area above the posterior third rib); compatible were those presenting a miliary pattern, pleural effusion or thoracic adenopathy, and atypical those showing any other abnormality [19].

### Case definition

PTB cases were defined as those with a positive culture for MTB in the respiratory specimen or those with clinical and radiological improvement after six months of solely anti-TB treatment, as judged by three different chest physicians in a blinded review, not involved in this study [20]. Non-PTB was considered in patients whose acid-fast smear and culture for MTB were negative and who had no chest radiographic changes after six months of follow-up. PCR results were not available for routine care or for the panel of experts.

Gold standard criteria for SNPTB final diagnosis included all PTB cases, confirmed or not by culture.

### Routine laboratory process

All sputum specimens were processed at the Public Reference Laboratory. All sputum specimens were tested by the Ziehl- Neelsen method, cultured in Lowentein Jensen and identified according to Kubica's method [21].

The presence of the amplified fragment derived from *IS*6110 insertion element sequence in PCRs positives was checked by electrophoresis with 2% agarose gel, stained with ethidium bromide, and visualized under ultraviolet light [16]. The positive and negative controls were included in electrophoresis analysis.

The PCR colorimetric dot-blot assay was performed as previously published [16]. Briefly, the biotinylated PCR products were transferred to a nylon membrane and hybridization was performed with a specific probe. The detection of hybridization was performed using a conjugated streptavidin-alkaline phosphatase probe. The positive reaction was obtained by adding BCIP and NBT (5-bromo-4-chloro-3-indoyl phosphate and nitro blue tetrazolium). The positive and negative controls were included for each set of PCR. To detect specimen inhibitors in negative results, a tube of PCR mix for each specimen was spiked with purified DNA target. All PCRs tests with discrepancies in results were tested in duplicate.

### Data analysis

Epidemiological and laboratory data were entered into a computer database and analyzed by appropriate statistical software (SPSS version 10.0). The endpoints were sensitivity (SE), specificity (SP), positive and and positive and negative predictive values (PPV, NPV) for detection in smear-negative subjects. For MTB DNA detection, the analyses of PCR SE, SP, PPV, NPV were performed on a per-study-subject basis, using the diagnosis of PTB as a reference standard (defined above). Agreement between the PCRs duplicates was evaluated using the Kappa score, a measurement of agreement that considers the excess of the amount of agreement that could be expected by chance.

For secondary analysis, using the high pretest probability (HPP) as a diagnostic test, suspects of SNPTB with high pretest probabilities were considered as positive for active PTB, and those with intermediate and low pretest probabilities were considered negative.

Additionally, test performances of HPP in parallel with PCR as a diagnostic test were calculated using specific formulas: SE of HPP with PCR: SE_HPP + _SE_PCR- _(SE_HPP + _SE_PCR_), predictive values (PV) for different prevalence rates according to the literature [22].

## Results

Of the total of 277 SNPTB suspects enrolled, 64 (23.1%) were not included in the analysis for the following reasons: 63 (22.6%) had an incomplete set of clinical data (14 patients had no chest X-ray available; 48 did not fulfill the SNPTB definition, one refused to participate, and the culture of one was contaminated. Of the 213 SNPTB suspects included in the analysis, all with known HIV test results, 104 (48.8%) were diagnosed with active TB. Stratifying by HIV status, the sensitivity of acid-fast bacilli in expectorated sputum was lower in HIV seropositive than in HIV seronegative suspects of SNPTB (67% vs 41%; p = 0.01). Among the 213 SNPTB suspects, 62 (29%) had positive smear acid-fast bacilli tests and were excluded. In this study, data were analyzed for 151 SNPTB suspects with negative smear tests. Overall, active PTB was diagnosed in 28.4% (43/151) of patients, HIV infection in 29.8% (45/151), and a history of previous PTB was referred to by 35.0% (53/151). Positive culture results occurred in 69.8% (30/43) of all TB cases, and 73.7% (14/19) of HIV seropositive TB cases.

### Analysis of pretest probability (clinical suspicion) for PTB

Clinical features of SNPTB suspects are shown in Table 1. In 151 SNPTB suspects, according to risk categories, the prevalence of PTB in high, intermediate and low clinical pretest probabilities was 67.4% (31/46), 24% (6/25) and 7.5% (6/80), respectively (p < 0.05). The proportion of patients with a suggestive chest radiograph increased steadily in those with a clinical suspicion of TB groups; 0% of low probability patients had suggestive radiographs, 24% in the intermediate group, and 78% of the high level group (p < 0.001).

**Table 1:** Patient symptoms and medical history, associated with physicians' clinical suspicion of tuberculosis among smear-negative PTB suspects

**Clinical Suspicion of Tuberculosis Group Smear-Negative PTB suspects**				
**Symptoms and Medical History**	**N = 151 (%)**	**Low (N = 80)**	**Intermediate (N = 25)**	**High (N = 46)**

Suggestive chest radiography^a^	42 (27.8%)	0	6	36
Weight loss	81 (53.6%)	37	12	32
Cough	135 (89.4%)	73	23	39
Chest pain	89 (58.9%)	48	15	26
Dyspnea	105 (69.5%)	57	17	31
Tuberculosis exposure at home	74 (49.0%)	37	14	23
Hospital admission in the last 24 months	53 (35.1%)	24	10	19
Hepatitis	31 (20.5%)	12	5	14
Immune suppression ^b^	51 (33.8%)	17	13	21

a: intake chest radiograph was suggestive of classical tuberculosis (upper-lobe fibrocavitary) disease.

b: includes patients positive for the human immunodeficiency virus (27.2%) and those with a history of steroid use or cancer chemotherapy (3.6%)

### Comparative performance analysis of tests

The performance of tests for detection of MTB and diagnosis of PTB are shown in Table 2. The PCR sensitivity was 65% (CI 95%, 50%–78%) and specificity was 83% (CI 95%, 75%–89%).

**Table 2:** Performance of Culture, PCR dot-blot and Clinical suspicion tests, individually and associated, in 151 smear negative PTB suspects

**Table 2A. Laboratory results and Performance of methods**									
		**All Group^a^** **N = 151**				**Non previously TB treated Group^b^** **N = 98**			
		
		**TB** N = 43		**Non-TB** **N **= 108		**TB** **N **= 36		**Non-TB** **N = 62**	

**Culture**	**Positive**	30		0		27		0	
	**Negative**	13		108		9		62	

		**SE**	**SP**	**PPV**	**NPV**	**SE**	**SP**	**PPV**	**NPV**
		
		70%	100%	100	89	75%	100%	100	87

**PCR dot-blot**	**Positive**	28		18		25		9	
	**Negative**	15		90		11		53	

		**SE**	**SP**	**PPV**	**NPV**	**SE**	**SP**	**PPV**	**NPV**
		
		65%	83%	61	86	69%	85%	73	83

**High PP**	**Positive**	31		15		26		4	
	**Negative**	12		93		10		58	

		**SE**	**SP**	**PPV**	**NPV**	**SE**	**SP**	**PPV**	**NPV**
		
		72%	86%	67	88	72%	93%	87	85

**Intermediate PP**	**Positive**	6		19		6		6	
	**Negative**	37		89		30		56	

		**SE**	**SP**	**PPV**	**NPV**	**SE**	**SP**	**PPV**	**NPV**
		
		14%	82%	24	71	17%	90%	50	65

**Low PP**	**Positive**	6		74		4		52	
	**Negative**	37		34		32		10	

		**SE**	**SP**	**PPV**	**NPV**	**SE**	**SP**	**PPV**	**NPV**
		
		14%	31%	7.5	48	11%	16%	7.1	24

**Table 2B. Performance of methods used in parallel**									

**Performance of PCR dot-blot in parallel with High Clinical Suspicion**		**SE**	**SP**	**PPV**	**NPV**	**SE**	**SP**	**PPV**	**NPV**
		90%	71%	75	88	91%	79%	81	90

**Performance of PCR dot-blot in parallel with Intermediate Clinical Suspicion**		**SE**	**SP**	**PPV**	**NPV**	**SE**	**SP**	**PPV**	**NPV**
		
		70%	68%	68	70	74%	77%	75	76

**Performance of PCR dot-blot in parallel with Low Clinical Suspicion**		**SE**	**SP**	**PPV**	**NPV**	**SE**	**SP**	**PPV**	**NPV**
		
		26%	25%	47	47	26%	14%	45	34

SE: Sensitivity, SP: Specificity, PPV: Positive Predictive Value, NPV: Negative Predictive Value, PP: pretest probability (clinical suspicion).

a: All group.

b: Non previously TB treated group patients

When the pretest probability (Clinical Suspicion) is used as a diagnostic test, the high pretest probability sensitivity was 72% and specificity was 86%. The Intermediary pretest probability sensitivity was 14% and specificity was 82%. The Low pretest probability sensitivity was 14% and specificity was 31%.

PCR had a similar sensitivity to the culture results (65% vs 70%, p = 0.65) and to the high pretest probability (65% vs 72%; p = 0.66). The PCR dot-blot demonstrated 18 false-positive results (9 had TB in the past, 1 presented a scar image in the chest X-ray that resembled inactive TB, 4 were HIV+, 4 referred proximity with smear positive PTB cases in the last 6 months). The PCR dot-blot demonstrated 15 false-negative results. The value of the Kappa score obtained between the duplicates of PCRs was 100%. PCR dot-blot inhibition was found in one SNPTB suspect (2.3%).

In a parallel evaluation, the sensitivity of PCR used in parallel with the high pretest probability was higher than the sensitivity of the high pretest probability when used alone (90% vs 72%), and of culture sensitivity (90% vs 70%) (Table 2B). PCR colorimetric dot-blot assay, used in parallel with the high pretest probability, had a PPV and NPV of 75% and 88%, respectively. The NPV was similar to that observed with culture alone (88% vs 89%). Comparing the SE and NPV of PCR used in parallel with the high pretest probability among those individuals not previously treated and those treated for TB in the past, the figures were respectively 91%, 90% vs 83%, 79%, p > 0.05.

In HIV seropositive subjects, the sensitivity of PCR was 63% (CI 95%, 40%–82%), and specificity was 85% (CI 95%, 66%–94%). The PCR sensitivity was similar to that of culture (74%) and to the high pretest probability method (74%). The PCR colorimetric dot-blot assay, used in parallel with the high pretest probability, had a SE, SP, PPV and NPV of 90%, 65%, 72%, and 88%, respectively.

In HIV seronegative subjects the sensitivity of PCR was 66%, and specificity was 83%. PCR sensitivity was similar to that of culture (67%) and to the high Pretest Probability method (71%). The PCR colorimetric dot-blot assay used in parallel with the high pretest probability had a SE, SP, PPV and NPV of 90%, 74%, 77%, and 89%, respectively.

Considering the HIV status and comparing the SE and NPV of PCR with high pretest probability among those individuals not previously treated and those treated for TB in the past, the figures were respectively: 91%, 90%, and 89%, 88% for HIV seropositive subjects and 93%, 92% and 83%, 80% for HIV seronegative group.

An increased sensitivity of PCR, when used in parallel with the high pretest probability, was observed in non-previously treated patients, compared to those who had had previous anti-TB treatment (sensitivity: 91% vs 83%). Similar results were found in HIV seropositive study subjects, in which the sensitivity was 91% among non-previously treated cases and 89% among those treated in the past. In HIV seronegative suspects, similar results were observed, in which sensitivity was 93% among non-previously treated cases and 83% among those treated in the past.

An increased specificity of PCR associated with a high pretest probability was observed in non-previously treated patients, compared to those who had previous anti-TB treatment (specificity: 79% vs 61%). Similar results were found in HIV seropositive study subjects: specificity was 79% among non-previously treated cases and 75% among those treated in the past. In HIV seronegative subjects, using the same diagnostic test, the specificity was 80% among non-previously treated cases and 62% among those treated in the past.

**Figure 1:**
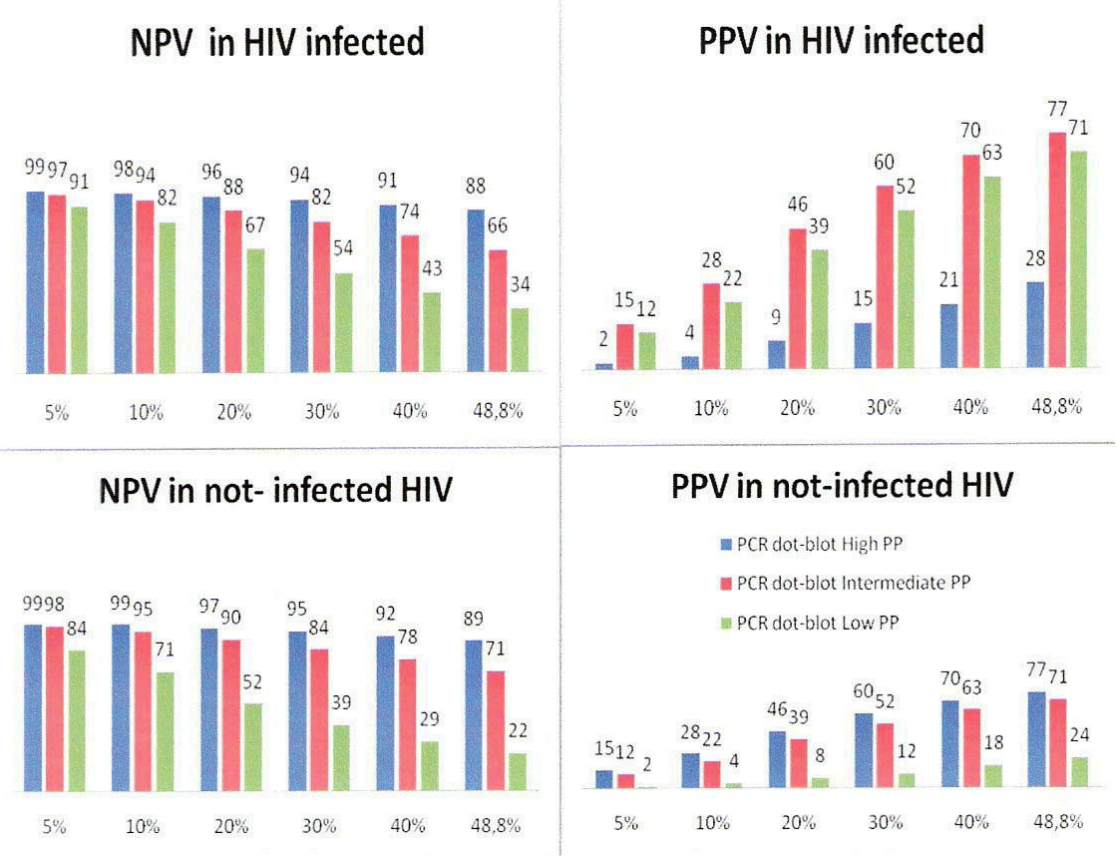
**Simulation of positive and negative predictive values of ZN and Culture/PCR dot-blot tests, according to different TB prevalence rates**. PPV: Positive Predictive Value, NPV: Negative Predictive Value, PP: pretest probability (clinical suspicion); TB prevalence rates: from 5% to 48.8%

Assuming different TB prevalence scenarios, the use of the PCR colorimetric dot-blot in parallel with a high clinical suspicion of SNPTB showed similar positive and negative PVs, among HIV seropositive and HIV seronegative patients (Figure 1). In regions from developing nations with a estimated TB prevalence of 5%–10%, described in out-patient units attending persons with coughs for more than three weeks (respiratory symptomatic, according to WHO), NPV for the PCR colorimetric dot-blot technique used in parallel with a high clinical suspicion of SNPTB, ranged from 98 to 99%, among HIV seropositive and HIV seronegative subjects. In Health Units, in which the prevalence ranges from 15% to 20%, usually in General Hospitals or Ambulatory Reference Centers (TB Clinics), negative PV of this diagnostic strategy ranged from 96% to 97%. In Reference TB Hospitals where the TB prevalence ranges from 30% to 40%, among HIV seronegative individuals, NPV of PCR colorimetric dot-blot, used in parallel with a high clinical suspicion of SNPTB, ranged from 95% to 92%, and among HIV seropositive individuals, this figure was of 94% and 91%, respectively (Figure 1).

The median time to reveal growth of MTB was 30 days (interquartile range [IQR] 30 to 45) for culture and the median time for detection of MTB by PCR was 3.32 days (IQR 3.0 to 3.75), respectively (p < 0.01). Within one week, of the 30 positive cultures, none were positive, whilst PCR detected 93% of positive specimens (p < 0.01).

## Discussion

We evaluated the performance of tests in SNPTB suspects. The strengths of this study, carried out in a developing country, included: a) a large number of SNPTB suspects, b) comparison were made according to HIV status, history of previous anti-TB treatment, the different levels of clinical suspicion and pretest probability and, c) the prospective design, ensuring a more complete clinical, laboratory, and radiographic information. Individuals were carefully characterized by independent reviews to determine the final diagnosis.

In this study, performed at the Hospital Reference Center, we observed a high prevalence of active PTB (48.8%) and TB/HIV co-infection (29.8%), confirming the epidemiological data described by the TB Control Program of Porto Alegre City, where 29% of all new TB cases reported yearly are diagnosed in hospitals and where there is a high TB and HIV burden [23].

The prevalence of PTB for the high pretest probability group was 67.4%, lower than that described in the literature where smear-positive and smear-negative PTB were included in analyses, the prevalence of PTB for intermediate pretest probabilities was 24%, similar to that (29%) described by Catanzaro, but higher than that described by Lim (3.4%). PTB prevalence for low pretest probability was 7.5%, higher than that cited elsewhere with low burdens of TB and HIV infection in health settings [24-26].

The moderate PCR sensitivity and specificity in SNPTB suspects (sensitivity: 65%; specificity: 83%) observed were similar to the sensitivities (61%–83%) and specificities (84%–92%) described by others [11,13,14]; however the specificities obtained were lower than those described in industrialized countries (>95%) [27]. As mentioned by others, in this report, the sensitivity of PCR was not influenced by the HIV status of the patient [12,28].

Decreased sensitivities of new diagnostic tests are expected among smear-negative pulmonary TB cases. In this study, this may be due to: 1) the presence of inhibitors that remain in the specimen after the extraction procedure; 2) a small number of mycobacteria unequally distributed in the test suspension; 3) a mycobacterium level that is below the detection limit of *in house *PCR (50 CFU) [16]. The proportion of inhibitors was 2.3%, and this result was lower than that reported by other studies using home-made PCR (22.7%) and similar to those using automated NAA (0.85%–5.0%) [17,27,29]. Twenty-three specimens presented less than 50 CFU in culture, which is below the detection limit of the test. Partial loss of mycobacterial homogeneity, leading to unequal distribution in the test suspension, may be due to the division of the suspension into three aliquots for use in laboratory tests. Additionally, a potential source of decreased sensitivity may be the use of the IS*6110 *insertion element as the target for PCR, since MTB can present low copy numbers of the element. Meanwhile, DNA fingerprinting studies, performed in Brazil, did not find the presence of these strains, as mentioned by Sperhacke *et al.*[16].

The decrease in specificity was due to eighteen false-positive results from patients that referred to previous anti-TB treatment, thus it is not surprising that DNA could be detected in their respiratory specimens, however the period elapsed between the end of previous anti-TB treatment in these patients and the reported positive PCR analyses was not collected, and this is a limitation of this study.

Using a high pretest probability as a diagnostic test for SNPTB diagnosis, this test had a sensitivity of 72% and a specificity of 86%; these values were similar to those observed with culture and PCR. These low accuracies can be explained by the fact that the evaluation of patients was performed by young or less experienced physicians, as described by LIM *et al.*[30]. The large disparity in sensitivities found when the clinical suspicion (pretest probability) is used as a diagnostic test, can be explained due to: a) the classification in risk categories based on based on clinical data, b) different years of experience in the SNPTB diagnosis of physicians that evaluated the patients. Therefore, when a high, intermediate and low probability pretest is used as a diagnostic test, patients with high risk had sensitivities higher than those of patients with intermediate and low risk, because these patients had more symptoms compatible with SNPTB.

The clinical evaluation used in parallel with the PCR test may be an alternative to the use of the PCR test for rapid diagnosis of PTB, especially in a hospital setting with a high burden of TB/HIV co-infection. The combination of clinical judgment and amplification results strongly enhances a rapid and correct diagnosis of PTB [26].

In this study, when we used PCR in parallel with high pretest probability, the diagnostic appeared to offer a higher negative predictive value in SNPTB subjects that had not been previously treated and in HIV seronegative cases, as described by others [24,25,30]. In non-previously treated and HIV seronegative cases, the performance results (SE: 93%; NPV: 92%) were similar to those recently described by Piersimoni et al, using automated tests in 214 PTB suspects [26].

Due to the small number of active TB in the group with the low pretest probability, additional evaluation is warranted in order to analyze the appropriateness of the parallel use of the PCR technique in this group of patients. However, the most difficult group for clinical assessment is the intermediate risk group, where PCR, used in parallel with the intermediary pretest (Clinical Suspicion), appears not to be useful, as already suggested by others. The prevalence of PTB may be overestimated in the intermediate risk group, thus the utility of PCR assay in these patients needs further evaluation, using more accurate clinical selection criteria [25,30].

The PCR dot-blot was selected due to its low cost (around U$12), simple extraction method and the colorimetric end point, all factors that might be expected to facilitate the transfer of NAA tests to laboratories in low income countries [28,31]. Additionally, as clinical risk assessment is more likely to reflect physician decision-making, to our knowledge, this is the first prospective study that relates pretest probability with the performance of a PCR in consecutive patients suspected of having SNPTB, in South America.

In our study, we pursued, in a large number of smear negative pulmonary TB suspects, a comprehensive clinical and laboratory approach for TB diagnosis using a home-made PCR, as suggested by Flores et al[32]. Due to the heterogeneity in the test's accuracy, it was emphasized the necessity to incorporate the clinical information for the better evaluation of NAAs in TB diagnosis among smear negative cases [32,33].

Another possible study limitation was the use of a home-made PCR that may warrant validation in comparison with more reliable techniques, such as an automated standardized test, as described by Greco *et al.*[33]. Unfortunately, an automated test was not available, therefore, the IS6110 element of insertion was used as a target for PCR; as recent meta-analyses demonstrated its higher accuracy in the diagnosis of SNPTB [11]. Differing data in literature may be explained due to some factors. Firstly, few studies have evaluated the utility of the home-made PCR technique among SNPTB suspects in developing nations with a high TB and HIV burden. Secondly, those studies that measured the clinical risk assessment were performed in settings with different TB prevalence [24,25]. Thirdly, clinical judgment and experience can influence the pretest probabilities, interfering with the sample size in each clinical risk group. Finally, the prevalence of co-morbidities (i.e.: HIV infection) of mycobacteria other than tuberculosis (MOTT) disease and the patient's response to interview may differ according to their prevalence in the community.

## Conclusion

The PCR dot-blot used in parallel with the high probability pretest has a high negative predictive value suggesting that in a hospital setting in developing countries, with a high prevalence of TB and HIV, the PCR technique may be useful for the evaluation of SNPTB suspects. For example, when the pretest probability is high, a negative PCR result indicates an increased likelihood of the absence of active TB in SNPTB suspects, infected or not by HIV.

We conclude that our results are in agreement with those of the literature, showing that molecular methods may provide an important contribution to the diagnosis of SNPTB in patients with high clinical suspicion [24,26]. Since home-made PCR is less costly than automated NAA, this test could be introduced more widely after a proper evaluation of its cost-effectiveness with clinical and radiographic characteristics to refine estimates of likelihood of TB disease in different settings, as proposed by others [28,34].

## Abbreviations

AFB – Acid Fast Bacilli

MOTT – Mycobacterium Other Than Tuberculosis

MTB – *Mycobacterium tuberculosis*

NAA – Nucleic Acid Amplification

PCR – Polymerase Chain Reaction

PTB – Pulmonary Tuberculosis

SNPTB – Smear Negative Pulmonary Tuberculosis

PRH – Parthenon Reference Hospital

TB – Tuberculosis

## Competing interests

The author(s) declare that they have no competing interests.

## Authors' contributions

LCS carried out the study, participated in the laboratory tests, participated in data acquisition, performed the statistical analysis and drafted the manuscript; RDS carried out the laboratory tests, data analysis, participated in data acquisition and drafted the manuscript, FCQM helped to draft the manuscript, CJ participated in the recruitment and in the clinical evaluation of patients, PIC underwent the questionnaire in patients. SM and MOR performed bacteriological tests, ARN performed the epidemiological analysis and drafted the paper, MLRR helped design the study, performed the statistical analysis and drafted the paper, ALK conceived of the study, participated in its design, performed the data analysis, coordination and helped to draft the manuscript. All authors contributed to the interpretation of results, have read and approved the final manuscript.

## Pre-publication history

The pre-publication history for this paper can be accessed here:


